# The 2008 *Retrovirology Prize*: Ben Berkhout and his RNA world

**DOI:** 10.1186/1742-4690-5-113

**Published:** 2008-12-11

**Authors:** Kuan-Teh Jeang

**Affiliations:** 1 the National Institutes of Health, Bethesda, MD, USA

## Abstract

Ben Berkhout wins the 2008 *Retrovirology Prize*.

## Editorial

Four years ago, *Retrovirology *inaugurated an annual prize to recognize the achievements of a deserving retrovirologist [1]. The *Prize *is supported in part by the Ming K. Jeang Foundation, a philanthropic charity based in Houston, Texas, which has provided for scholarships at Houston schools, at the University of Arizona, and at the Johns Hopkins University School of Medicine. Previous winners of the *Retrovirology Prize *include Stephen Goff [2], Joseph Sodroski [3], and Karen Beemon [4]. A goal of the *Retrovirology Prize *is to identify an outstanding mid-career scientist who is close to the peak of his/her productivity and who is expected to have many future years of high achievement.

**Figure 1:**
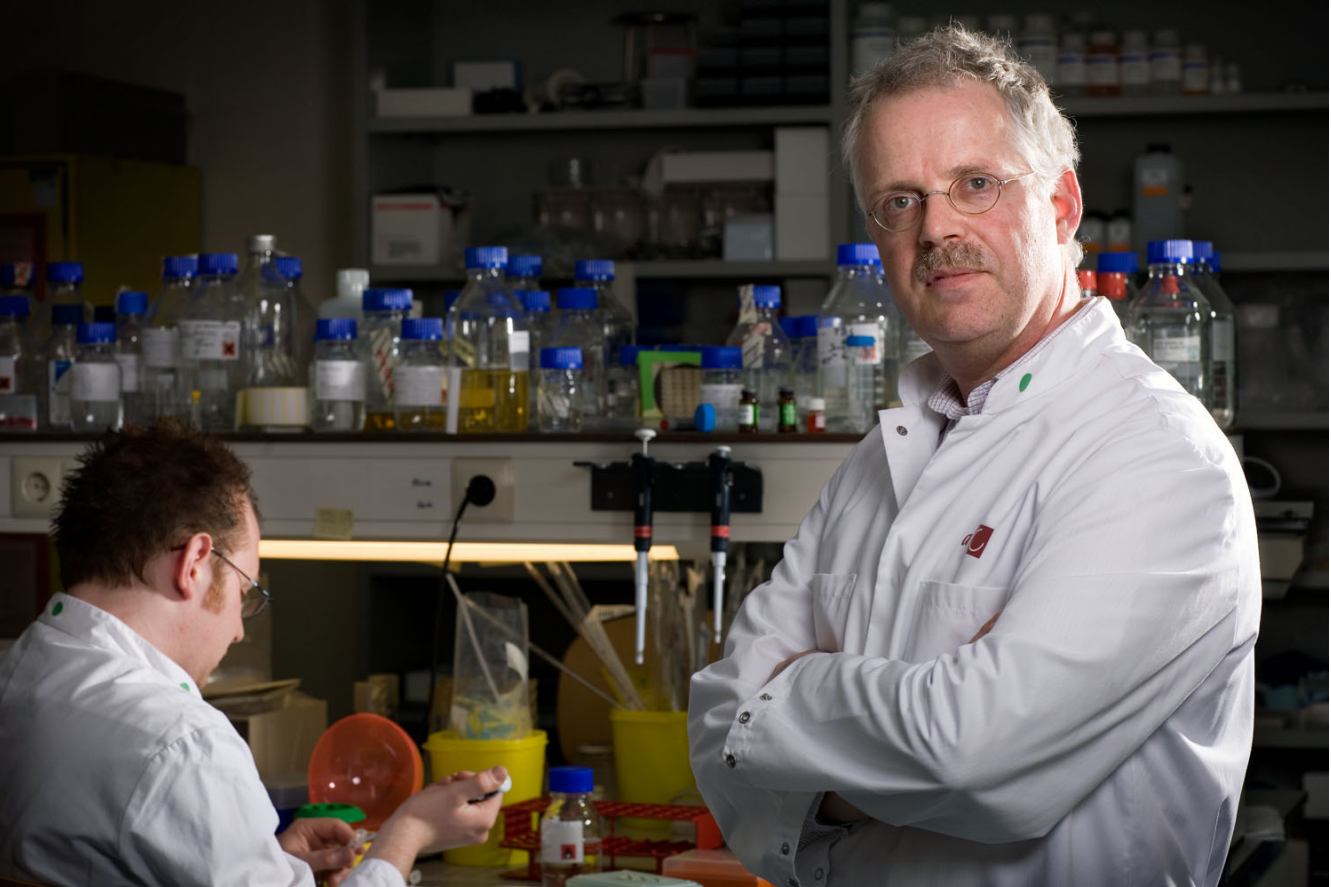
A picture of Ben Berkhout, Professor and Head of the Laboratory of Experimental Virology at the Academic Medical Center (AMC) of the University of Amsterdam, the Netherlands, in his laboratory.

For 2008, the Editors of *Retrovirology *selected Ben Berkhout as the recipient of the *Retrovirology Prize *(Figure 1). Dr. Berkhout is Professor and Head of the Laboratory of Experimental Virology at the Academic Medical Center (AMC) of the University of Amsterdam, the Netherlands. In the late 1980s, he was instrumental in changing the paradigm of thinking on gene regulation. At a time when the focus in eukaryotic transcription was on DNA-enhancers and DNA-binding proteins, Berkhout emerged to propose HIV-1 TAR RNA and the viral Tat protein as prototypes of RNA and RNA-binding protein mediated mammalian gene regulation [5]. That novel insight, focusing on TAR as RNA rather than DNA, subsequently directed efforts toward the cloning of additional TAR RNA-binding proteins, such as the human TRBP protein [6], which is now recognized to play a critical role in interferon signaling, RNA interference, and micro-RNA biogenesis.

Dr. Berkhout's research on RNA has provided additional important building blocks for many other aspects of our current knowledge on HIV-1 replication. He has employed a multi-disciplinary approach to research, combining methods from molecular biology, biochemistry and most importantly, virology. His research has extended our insights into the mechanisms of transcription, reverse transcription, drug-resistance, and RNA interference. For example, Berkhout and his colleagues have been instrumental in the identification of the primer-activating signal (PAS) for reverse transcription, the description of the fitness defects in drug-resistant HIV-1 variants, the first description of the *in vivo *evolution of a drug-dependent HIV-1 variant under therapy pressure, the characterization of the HIV-1 polyA hairpin structure and mechanisms for regulating polyadenylation, the elucidation of novel HIV-1 escape routes from RNAi-inhibition, and the construction of a conditionally-live HIV-1 variant as novel vaccination strategy, amongst other findings.

To understand Ben's career development and his motivations in science, I had an opportunity to converse with him regarding his views on several questions. His responses to a dozen of my queries are below.

**KTJ: **Today's young people consider many other careers to be more attractive than science. Tell us a bit about what motivated and attracted you to science?

**BB: **During my chemistry study at the University of Leiden, I performed a traineeship in the Biochemistry department where they worked on the mechanism of mRNA translation in Escherichia coli. This was old-fashioned biochemistry, ribosome isolations on Monday morning in the cold room etc. It was fascinating to me that by mixing several ingredients in a test tube you could learn something about this invisible process of nature. A little later the introduction of molecular biology techniques greatly accelerated this field, and our favorite model system was the RNA bacteriophage MS2, one of the first viruses for which an infectious cDNA clone had been generated. I also performed my thesis work in Leiden, with Jan van Duin, learning a few molecular RNA-tricks that are used by the MS2 virus to regulate its gene expression.

**KTJ: **You didn't start by studying HIV; what changed your mind during your postdoc days to turn to this field?

**BB: **I received a 1-year fellowship from the Dutch Cancer Society to perform post-doctoral research at the Dana Farber Cancer Institute of the Harvard Medical School. I stayed there almost 3 years and learned a lot on the topic of T cell immunology, but slowly came to realize that I was missing RNA molecules, and viruses in particular. I guess immunology remains too descriptive for me; at the end of the day I want to learn something in molecular terms that no one else knows. After a focused search, I decided in 1988 to move into the HIV-1 field and ended up in the laboratory of Kuan-Teh Jeang, by then a young group leader at the National Institutes of Health in Bethesda. That was the start of a very exciting and productive period. After a 5-year stay in the USA, my HIV-1 research was continued at the University of Amsterdam, where I still reside.

**KTJ: **You are perhaps best known for your paper on TAR as a nascent RNA target for Tat [5]. Walk us through on your thinking at the time and what factors influenced your thought?

**BB: **The expertise of the laboratory was on HIV-1 and HTLV-I, but mostly on the process of transcriptional regulation by the LTR promoters. The TAR RNA hairpin motif immediately attracted my interest, and the initial mutants I generated were the ones that ended up in that 1989 Cell paper. In fact, I designed a way to specifically mutate structured RNA signals and not the underlying DNA sequences, an idea that is largely based on a natural mechanism of regulated gene expression in bacteria that was worked out by Charles Yanofsky at Stanford University. This method also allows you to address kinetic aspects, and that is how we ended up talking about the nascent RNA transcript. We did not dare to use the word "RNA enhancer" in that paper, but this term was subsequently coined by Phil Sharp in an accompanying commentary. Looking back, the combination of my RNA expertise and the transcriptional focus of the lab turned out to be key in this break-through. We have since kept an interest in the functions of the TAR motif and the Tat protein.

**KTJ: **Who are the scientists who have influenced your career and how?

**BB: **Jan van Duin taught me how to design experiments and how to think carefully and critically about the generated data. Kuan-Teh Jeang guided me on how to be incisive and how to present results in writing and at meetings. But in fact, many people have shaped my thinking and the direction of my research.

**KTJ: **HIV research has been going on for more than a quarter century, and a Nobel prize was just awarded for the discovery of HIV-1. What do you see as the most important question still facing HIV-1 research and what are your thoughts on why we haven't made better progress?

**BB: **The one thing that is badly needed is an effective and cheap vaccine that protects against HIV-1 transmission. It is clear that there are currently no vaccine candidates that look promising, and we should thus maximize the available possibilities for prevention. It is obvious that the relatively simple vaccination strategies that work for other pathogens do not work for HIV-1. Despite all the funds that have been available for HIV-1 vaccine research, it is also fair to say that only a few approaches have been seriously tried. The failure of these initial attempts, that is the envelope protein and adenovirus-based vaccines, has recently led to the suggestion to diversify our vaccination approaches. I could not agree more, and we should be on the lookout for young researchers with fresh ideas.

What went wrong? This is an interesting question that may provide us with some important lessons for the future. It may be that a "too early" focus on a certain vaccine candidate, which in itself is worth testing but certainly not worth putting all the money on. In fact, I think that this is a particular problem of a densely populated research area such as the HIV-1 field. The scientific crowd usually follows a few leaders, without too much critical thinking, and it may mean that momentum is built around a track that turns out to be the wrong one. Diversification and special focus on researchers that are new to the field will be important. We have a few unique vaccine projects running in the lab. I am not saying they will be successful; it is all high-risk, but they are for sure different from mainstream approaches. Let's hope that one of these "crazy" ideas will lead the way to that protective vaccine.

**KTJ: **You have been an editor of *Retrovirology *for the past 5 years. What do you see in the future for Open Access publishing and what do you consider as the advantages/disadvantage of Open Access versus the traditional subscription based publishing?

**BB: **From the start, I truly liked the basic idea that results presented in an Open Access journal are available to the reader anywhere on the globe without any financial or other barrier other than access to the internet. A major motivation for most authors to publish in an Open Access journal is increased visibility and ultimately a citation advantage. There is some research indicating that Open Access articles are cited more frequently or earlier than non-Open Access articles. I do not see any major disadvantage, and note that some of our traditional journals have successfully moved to the Open Access format (e.g. *Nucleic Acids Research*). The future is with Open Access journals.

**KTJ: **You have published several notable papers in *Retrovirology *[7-11]. How did you decide to publish in *Retrovirology*? For the future direction of this journal, what would you try to improve?

**BB: **As member of the editorial board of *Retrovirology*, we of course wanted to present some of our own studies in this new journal, although in the beginning *Retrovirology *like all upstart journals was without an official impact factor. But, it was rewarding to see that when the first official impact factor for *Retrovirology *was released it was a very respectable number, 4.04 in 2007. This number ranked the journal second on the Impact Factor list for virology journals that publish original research articles (*Retrovirology *ranks behind only the *Journal of Virology*). Indeed, over the last few years, virologists around the world have increasingly embraced *Retrovirology*, and we now receive many more submissions than we can publish. I look forward to further enhancing the quality threshold needed for publishing in *Retrovirology*.

**KTJ: **We are perhaps at a tipping point in the globalization of science. There are some predictions that by 2040, China and India will be in the top three of world economic powers. How (what) do you think that American and European science should do (more) to engage science/scientists from the emerging economies?

**BB: **Science is of course an international activity and we usually meet our colleagues from all over the world at meetings, and many promising collaborations do exist. However, more should be done in particular with respect to the training of young scientists. We are used to the (usually transient) brain drain of European post-docs to the USA, but one should recognize the importance of such training programs on a worldwide scale, and going in all directions. A continuous dialogue between Western and Eastern countries (and others) is very important to appreciate the differences in cultural background that underlie differences in perception of scientific issues, and to start building bridges from there.

**KTJ: **Several recent articles have been written about the aging of Western scientific leadership and the difficulties confronted by younger scientists in garnering support. What are your thoughts on these issues?

**BB: **The aging issue in itself should not be a problem, as I know many sharp scientists with grey hair. Being a mid-career scientist, at least according to the rules of the *Retrovirology Prize*, I do indeed realize that it is of utmost importance to facilitate the career of promising younger colleagues. In my own laboratory, I have witnessed over the last few years that younger colleagues do take opportunities provided to them to lead and supervise individual studies. For these studies, they should claim and do become the last (senior) author for the published papers. This is the correct thing to do, and senior authorship is a critical factor in their attempts to compete for funding. We should get rid of the automatic last authorship for professors and departmental heads that usually lasts till their retirement or even thereafter. Special funding possibilities for young scientists are obviously important. We have such a funding scheme in the Netherlands for promising post-docs (Veni-Vidi-Vici program), and the EU has the Marie Curie fellowship program.

**KTJ: **One of the goals of the *Retrovirology Prize *is to promote the visibility of an outstanding mid-career scientist so that he/she could do more over the next few years. What are the next big ideas and projects that you would like to pursue?

**BB: **We have over the years built a rather big research lab with a core of basic HIV-1 studies and a few exciting applied projects. The vaccine candidate that is based on a conditionally replicating HIV-1 variant is now in macaque studies (in a SIV version) and our antiviral gene therapy based on RNA interference has recently moved into a pre-clinical humanized mouse model. Thus, we have exciting times ahead of us. We obviously will also continue the basic HIV-1 replication studies, with a focus on understanding bits and pieces of the structure and function of the HIV-1 RNA genome. Last but not least, we will continue some of the intriguing HIV-1 evolution studies that my lab is well-known for. This theme fits with 2009 as the bicentennial of Charles Darwin's birth and the 150th anniversary of the publication of his seminal work 'On the origin of Species'.

**KTJ: **In my office there is a quotation by Edmond Burke 'All that is necessary for the triumph of evil is that good men do and say nothing'. If there is one thing that you think needs to be said or done in science (or in society at large), what would that be?

**BB: **It is obvious that large-scale approaches to scientific discovery have arrived over the recent years, and these are particularly powerful to address certain research questions. There is also a trend among funding bodies to specifically ask for large, multidisciplinary consortia. I am not saying these recent trends are necessarily bad, but one should not forget the individuals and smaller groups with a strong track record, as they are frequently key in big discoveries.

**KTJ: **With the understanding that it will be a long time in the future, what would you like your headstone to read?

**BB: **How about "He was privileged to be paid to execute his hobby"?
